# Genome-wide transcription profiling of human sepsis: a systematic review

**DOI:** 10.1186/cc9392

**Published:** 2010-12-29

**Authors:** Benjamin M Tang, Stephen J Huang, Anthony S McLean

**Affiliations:** 1 Department of Intensive Care Medicine, Nepean Hospital and Nepean Clinical School, University of Sydney, Penrith, NSW 2750, Australia; 2 School of Public Health, Faculty of Medicine, University of Sydney, NSW 2006, Australia

## Abstract

**Introduction:**

Sepsis is thought to be an abnormal inflammatory response to infection. However, most clinical trials of drugs that modulate the inflammatory response of sepsis have been unsuccessful. Emerging genomic evidence shows that the host response in sepsis does not conform to a simple hyper-inflammatory/hypo-inflammatory model. We, therefore, synthesized current genomic studies that examined the host response of circulating leukocytes to human sepsis.

**Methods:**

Electronic searches were performed in Medline and Embase (1987 to October 2010), supplemented by additional searches in multiple microarray data repositories. We included studies that (1) used microarray, (2) were performed in humans and (3) investigated the host response mediated by circulating leukocytes.

**Results:**

We identified 12 cohorts consisting of 784 individuals providing genome-wide expression data in early and late sepsis. Sepsis elicited an immediate activation of pathogen recognition receptors, accompanied by an increase in the activities of signal transduction cascades. These changes were consistent across most cohorts. However, changes in inflammation related genes were highly variable. Established inflammatory markers, such as tumour necrosis factor-α (TNF-α), interleukin (IL)-1 or interleukin-10, did not show any consistent pattern in their gene-expression across cohorts. The finding remains the same even after the cohorts were stratified by timing (early vs. late sepsis), patient groups (paediatric vs. adult patients) or settings (clinical sepsis vs. endotoxemia model). Neither a distinctive pro/anti-inflammatory phase nor a clear transition from a pro-inflammatory to anti-inflammatory phase could be observed during sepsis.

**Conclusions:**

Sepsis related inflammatory changes are highly variable on a transcriptional level. We did not find strong genomic evidence that supports the classic two phase model of sepsis.

## Introduction

Sepsis is characterised by a bewildering array of abnormalities in both innate and adaptive immune systems. To help explain this complex pathophysiology, a two-phase model has been used by investigators. This model postulates that sepsis consists of an initial phase of systemic inflammatory response syndrome, followed by a later phase of compensatory anti-inflammatory response syndrome. This two-phase model has been the reigning paradigm under which scientists develop new therapeutic agents, with new drugs targeting either the pro-inflammatory or the anti-inflammatory arm of the host response. However, clinical trials have consistently failed to demonstrate any survival benefit of drugs that target the inflammation pathway. As a result, concerns have been raised regarding the validity of treating sepsis simply as a pro-inflammatory or anti-inflammatory phenomenon.

Complicating this uncertainty is the limited evidence to verify the two-phase model. Cytokine studies have been the mainstay evidence that provide support for the inflammation-based model. However, increasingly conflicting findings have emerged from recent cytokine studies [1-3]. Furthermore, it is often difficult to determine the exact nature of the host response (for example, pro-inflammatory versus anti-inflammatory) on the basis of cytokine measurement alone, which is highly variable depending on the choice of the cytokine used and the timing of the measurements.

Given the limitations of the protein level studies, we assessed the validity of the inflammation-based model using transcriptional level data. Genome-wide transcriptional studies have recently emerged as a powerful investigational tool to study complex disease [4]. These studies avoid the selection bias inherent in most cytokine studies, where only a small number of pre-selected genes can be examined. In this systematic review, we synthesized genomic data of recent microarray studies where the transcriptional changes of circulating leukocytes were examined in both experimental and clinical sepsis in humans.

## Materials and methods

### Search strategy and selection criteria

We searched in Medline and Embase, without language restriction, all publications on gene-expression studies between January 1987 and October 2010. In 1987 DNA array technology was first described, hence this year formed the starting point of our search [5]. We hand-searched the reference lists of every primary study for additional publications. Further searches were performed by reviewing journal editorials and review articles.

The search strategy used the following search terms: (1) "gene-expression profiling", (2) "microarray analysis", (3) "transcription profiling", (4) "cluster analysis", (5) "Affymetrix", (6) "GeneChip", (7) "sepsis", (8) "sepsis syndrome", (9) "septicaemia", (10) "bacteraemia", (11) "septic shock", (12) "infection", (13) "systemic inflammatory response syndrome", (14) "SIRS", (15) "systemic inflammation", (16) "endotoxin".

We also performed searches in public repositories of microarray datasets, including the National Centre for Biotechnology Information (Gene Expression Omnibus), the European Bioinformatics Institute (ArrayExpress), and the Centre for Information Biology Gene Expression Database (CIBEX). Datasets from microarray database were then cross-referenced with publications retrieved from Medline and Embase. Only datasets published as full reports were included in the final analysis.

We included a broad spectrum of gene-expression studies, including ones that are (1) cross-sectional or longitudinal design, (2) on different microarray platforms, (3) on whole blood or purified leukocytes, (4) in healthy volunteers or infected human hosts, and (5) paediatric or adult patients. As we only sought data on a genome-wide scale, we have excluded studies that assayed only a small number of genes, such as (1) Northern blot or PCR, (2) single gene or individual pathway studies, (3) proteomic studies, and (4) single-nucleotide polymorphism studies. We included custom designed microarrays only if such arrays are designed to study changes in inflammation pathways. Since we were interested in host response on a systematic level, as reflected by circulating leukocytes, we have excluded studies that (1) focused on resident immune cells such as alveolar macrophages or lymphoid tissue cells, and (2) used solid organ tissues such as spleen or liver.

### Data extraction

We extracted study level data according to a pre-specified template, which included participant demographics, country of origin, clinical setting and inclusion criteria. A separate template was used to collect details of microarray experiments, including sample collection procedures, cell separation techniques, target cell types, methods used to extract ribonucleic acids, cDNA synthesis and hybirdization, microarray platforms used, number of probe set on arrays, microarray data processing and normalization methods. We extracted the signature gene list from each published report or from the accompanied data file in the journal websites. Where available, results of functional analyses were also extracted. These included results of cluster analyses, principle component analyses or pathway analyses.

### Quality assessment

We performed a quality assessment of each study based on criteria modified from published guidelines on the statistical analysis and reporting of microarray data [6]. The assessment was performed using a 14-item checklist covering three quality domains including data acquisition (three items), statistical analysis (six items) and validation of microarray findings (five items).

### Data synthesis

We performed a narrative synthesis on genomic data extracted from each study. First, individual genes from the gene list of primary studies were manually annotated by cross-referencing with publicly available gene nomenclatures databases (for example, Genebank, Locuslink, Affymetrix gene identifiers). Where a gene list was not available, findings on functional analyses reported by the original authors were used. These included cluster analysis or gene network analysis performed on the original microarray data. All results were then collated and presented in evidence tables. Due to the heterogeneous nature of the included studies, meta-analysis of the microarray data was not performed.

## Results

**Figure 1:**
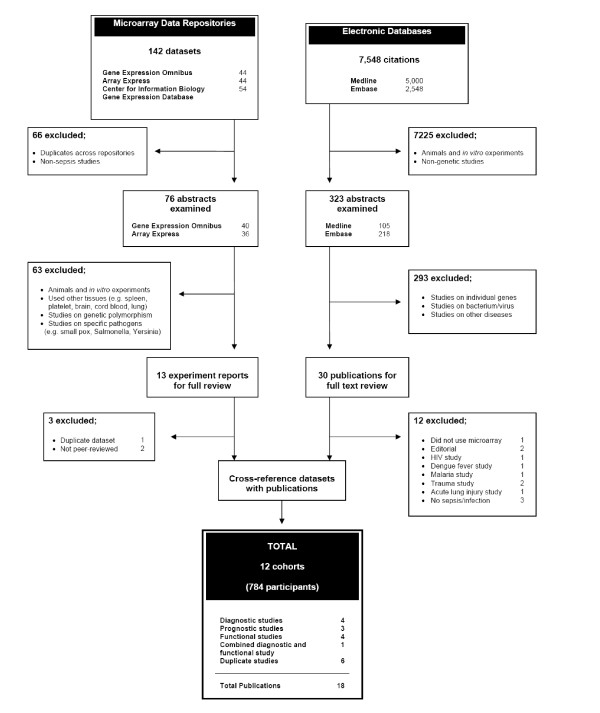
**Study selection**.

The literature search yielded 7,548 citations in electronic databases and 142 datasets in microarray data repositories. Of these, 12 patient cohorts met the inclusion criteria and were included in the final analysis (Figure 1).

Clinical characteristics of the included studies are summarized in Table 1. The cohorts were drawn from a broad spectrum of clinical settings including hospital wards, intensive care units and university research centres. The majority of the study participants were critically ill patients diagnosed with sepsis or infection. Among patients with sepsis, a full range of sepsis syndrome was represented (for example, sepsis, severe sepsis and septic shock).

**Table 1:** Summary of studies characteristics

	**Prucha **[14]	**Tang-1 **[15,16]	**Ramilo **[17]	**Tang-2 **[18]	**Talwar **[8]	**Payen **[19]	**Cobb **[20,21]	**Pachot **[22]	**Prabhakar **[9]	**Calvano **[10]	**Wong **[23-26]	**Johnson **[27,28]
**Aims**	Diagnostic prediction	Diagnostic prediction	Diagnostic prediction	Diagnostic prediction	Functional analysis	Prognostic study	Prognostic study	Prognostic study	Functional analysis	Functional analysis	Combined analysis^¥^	Functional analysis
**Study design**	Cross-sectional	Cross-sectional	Cross-sectional	Cross-sectional	Longitudinal	Longitudinal	Longitudinal	Cross-sectional	Longitudinal	Longitudinal	Longitudinal	Longitudinal
**Country**	Czech Rep..	Australia	U.S.A.	Australia	U.S.A.	France	U.S.A.	France	U.S.A.	U.S.A.	U.S.A.	U.S.A.
**Total (n)**	12	94	148	70	12	17	176	38	12	14	101	90
**Mean Age (yr)**	58.9	63.5	3.4	65.5	30	59	35.7	67	(18 to 40)^†^	(18 to 40)^†^	3.2	44
**Clinical setting**	Adult ICU	Adult ICU	Pediatric wards	Adult ICU	University clinic	Adult ICU	Adult ICU	Adult ICU	University clinic	University clinic	Pediatric ICU	Trauma ICU
**Inclusion criteria**	Severe sepsis	Sepsis	Acute infection	Sepsis	Healthy volunteers	Septic shock	Post-trauma	Septic shock	Healthy volunteers	Healthy volunteers	Sepsis	SIRS
**Control group**	Surgical patients	SIRS patients	Healthy subjects	SIRS patients	Healthy subjects	Subjects at time zero	Non-septic patients	NA	Subjects at time zero	Healthy subjects	Non-septic patients	SIRS patients

SIRS denotes systemic inflammatory response syndrome. ICU denotes intensive care unit. NA denotes not applicable.

^†^Mean age not available. ^¥^Both functional analysis and diagnostic prediction.

Details of the microarray experiments are summarized in Tables 2. The target tissue was either whole blood or purified leukocytes isolated from whole blood (for example, neutrophils or mononuclear cells). Affymetrix was the most common microarray platform used. In total, gene-expression profiling of 784 individuals were performed across four different microarray platforms.

**Table 2:** Microarray experiments in included studies

	**Prucha **[14]	**Tang-1 **[15,16]	**Ramilo **[17]	**Tang-2 **[18]	**Talwar **[8]	**Payen **[19]	**Cobb **[20,21]	**Pachot **[22]	**Prabhakar **[9]	**Calvano **[10]	**Wong **[23-26]	**Johnson **[27,28]
**Experiment details**												
**Tissue used**	Whole blood	Neutrophils	PBMC	PBMC	PBMC	PBMC	PBMC	Whole blood	PBMC	Whole blood	Whole blood	Whole blood
**RNA extraction**	PAXGene	Ambion	Qiagen	Ambion	Qiagen	Qiagen	Qiagen	PAXGene	Qiagen	Qiagen	PAXGene	PAXGene
**Microarray platform**	Lab-Arraytor	In-house	Affymetrix	Affymetrix	Affymetrix	Lab-Arraytor	Affymetrix	Affymetrix	In-house	Affymetrix	Affymetrix	Affymetrix
**No. of genes or probe sets**	340	18,664	14,500	54,675	12,623	340	54,613	14,500	18,432	33,000	54,675	54,675
**Signature genes**												
**Sepsis vs. control**	50	50	137	138	867		1,837		54	3,714	1,906	459
**Survival vs. death**						10		28				

^¶^Signature genes were searched but not found.

RNA denotes ribonucleic acid. G-Pos/Neg denotes Gram-Positive sepsis or Gram-Negative sepsis. PBMC denotes peripheral blood mononuclear cells.

Results on the assessment of the methodological quality of each microarray study are presented in Table 3. Just over half of the studies fulfilled the MIAMI criteria (Minimum Information About Microarray Experiment, published guidelines on the design, conducting, analysis and reporting of the microarray experiments) [7]. Only seven studies performed internal validation of microarray data and independently validated their reported gene lists in separate data sets. Raw microarray data are available in only 7 out of the 12 cohorts.

**Table 3:** Methodological quality of microarray experiments

	**Prucha **[14]	**Tang-1 **[15,16]	**Ramilo **[17]	**Tang-2 **[18]	**Talwar **[8]	**Payen **[19]	**Cobb **[20,21]	**Pachot **[22]	**Prabhakar **[9]	**Calvano **[10]	**Wong **[23-26]	**Johnson **[27,28]
**Data acquisition**												
Tissue homogeneity of target samples	Low	High	High	High	High	High	High	Low	High	Low	Low	High
Experiments follow miame criteria^¶^	Yes	Yes	Yes	Yes	Not clear	Yes	Not clear	Not clear	Not clear	Not clear	Yes	Not clear
Reporting of normalization method	No	Yes	Yes	Yes	Yes	Yes	Yes	Yes	No	Yes	Yes	Yes
**Analytical issues**												
Method for gene selection	*t *test	*t *test	Non-parametric test	*t *test	ANOVA	*t *test	Multiple	Not clear	Not clear	SAM	ANOVA and fold change	Non-parametric test
Issue of variance estimation addressed	No	Yes	No	Yes	No	No	Not clear	Not clear	Not clear	Yes	No	No
Comparison to other diagnostic markers	No	No	No	No	No	NA	Yes	Yes	No	No	No	Yes
Correction for multiple testing	Yes	Yes	Yes	Yes	Yes	NA	Yes	Yes	No	Yes	Yes	Yes
Reporting of classifier performance	No	Yes	No	Yes	NA	NA	No	Yes	NA	NA	Yes	NA
Reporting of prediction accuracy	No	Yes	Yes	Yes	NA	NA	Yes	Yes	NA	NA	Yes	NA
**Validation of data**												
Cross validation of signature genes	No	Yes	Yes	Yes	No	No	Yes	Yes	NA	No	Yes	Yes
External validation in independent samples	No	Yes	Yes	Yes	No	No	Yes	Yes	NA	Yes	Yes	No
Ratio of test/training sample size	NA	1.14	2.00	1.00	NA	NA	0.50	0.23	NA	0.75	0.77	NA
Adjustment for confounders	No	Yes	Yes	No	NA	NA	No	No	No	NA	Yes	Yes
Raw data made publicly available	No	Yes	Yes	Yes	Yes	Yes	No	No	No	No	Yes	No
PCR validation	Yes	No	Yes	Yes	Yes	Yes	Yes	Yes	Yes	No	No	Yes

Minimum Information About Microarray Experiment checklist [7]. ANOVA denotes analysis of variance. SAM denotes Significance Analysis of Microarrays [29].

NA denotes not applicable.

A wide range of statistical approaches were used by the included studies. Table 3 provides detailed information on the reporting of the statistical methods by each study. Most studies provided details on the method used for normalization. Normalization is a data processing method that ensures only genes, which are truly differentially expressed between phenotypes of interest, are detected, instead of those caused by experimental artefacts or variation in the microarray hybirdization process. Different statistical approaches were used for detecting statistically significant genes, depending on the study design used in each cohort (Table 3). Multiple testing corrections were used by most studies to minimize a false positive rate in the significant genes (Table 3). However, variance estimation was poorly reported in most studies. A variety of variance estimation techniques were used by the included studies; but details were lacking in most studies (conventional *t*-statistics based variance estimation methods under-estimate the true variance of microarray data, so several variance estimation methods for microarray data have been developed). Overall, the reporting of statistical methods was variable among studies.

### Pathogen recognition

Sepsis activates pathogen recognition pathways in host leukocytes. This is evident in most studies. Up-regulation of pathogen recognition receptors, such as toll-like receptors and CD14, was observed (Table 4). This was accompanied by the activation of signal transduction pathways, a process essential for subsequent transcription of immune response genes. The signal transduction pathways include nuclear factor kappa-B (NK-kβ), mitogen activated protein kinase (MAPK), Janus kinase (JAK) and transducer and activator of transcription protein (STAT) pathways (Table 4). The up-regulation of both pathogen recognition and signal transduction pathway genes was observed in most cohorts, including experimental and clinical sepsis, paediatric and adult patients, early and late sepsis.

**Table 4:** Gene-expression changes in pathogen recognition

	Pathogen recognition	Signal transduction
**Johnson **[27,28]	Increase expression in toll-like receptor (TLR) pathway genes.	Increased expression in pathways genes associated with NF-*k*B, STAT, JAK and MAPKs.
**Talwar **[8]	Increase expression in TLR pathway genes.	Increased expression in genes associated with STAT, JAK and MAPKs pathways.
**Calvano **[10]	Increase expression in TLR pathway genes and CD14 genes.	Increased expression in genes associated with STAT, NF-*k*B, CREB, JAK and MAPKs pathways.
**Prabhakar **[9]	Increase expression in genes encoding for CD14 molecules.	Increased expression in genes associated with JAK pathway.
**Prucha **[14]		Increased expression in genes associated with MAPKs pathway.
**Tang-1 **[15,16]		Reduced expression in pathways genes associated with NF-*k*B and MAPKs pathways.
**Tang-2 **[18]	Increase expression in TLR pathways genes.	Increased expression in genes associated with JAK, STAT and MAPKs pathways.
**Cobb **[20,21]		Increased expression in genes associated with MAPKs pathway.
**Wong **[23-26]	Increase expression in TLR pathways genes.	Increased expression in genes associated with NF-*k*B STAT and MAPKs pathways.
**Payen **[19]	Increase expression in TLR pathways genes in survivors.	Greater expression of genes associated with MAPKs pathway in non-survivors.
**Pachot **[22]	Increase expression in TLR pathways genes in survivors.	Greater expression of genes associated with MAPKs pathway in non-survivors.

**Abbreviations**; NF-ĸB denotes nuclear factor kappa-B, MAPKs denotes mitogen activated protein kinase, JAK denotes Janus Kinase, STAT denotes transducer and activator of transcription protein, CREB denotes cAMP responsive element binding protein, TLR denotes toll-like receptor.

### Inflammatory response

In contrast to the above findings, changes in inflammatory pathways were much less consistent. A distinctive pro-inflammatory or anti-inflammatory phase, as depicted in the classic sepsis model, was not seen during any stage of sepsis. The early, transient rise in some pro-inflammatory mediators was evident only in a minority of studies (Table 5). In some studies, the expression of anti-inflammatory genes dominated over pro-inflammatory genes. In others, changes in inflammatory genes were noticeably absent. No studies demonstrated a clear transition from a pro-inflammatory phase to an anti-inflammatory phase during the course of sepsis. Overall, the transcriptional changes in inflammation-related genes are highly variable in most cohorts.

**Table 5:** Gene-expression in inflammation and immunity

	Timing	Gene-expression	Overall effect	Changes in inflammatory and immune genes
**Johnson **[27,28]	Pre-sepsis (12 to 36 hrs prior to the diagnosis)	↑394 genes and ↓65 genes	Activation of host response to infection.	Increased expression of genes associated with pro-inflammatory cytokines (IL-1, IL-18), immune cell receptor signalling (IFNR, IL-10RA, TNFSF) and T cell differentiation (IFNGR, IL-18R, IL-4R).
			Activation of counter-regulatory mechanism that limits the pro-inflammatory response.	Increased expression of genes that limit pro-inflammatory cytokines (SOCS3).
**Talwar **[8]	Early Sepsis (0 to 24 hrs)	↑439 genes and ↓428 genes	Activation of host response to infection.	Increased expression of genes associated with cytokines (IL-1R, CCR1, CCR2, IL-17) and S100 calgranulins (S100A12, S100A11, S100A9, S100A8). Increased expression of genes associated with arachidonate metabolites (ALOX5) and anti-pathogen oxidases (CYBA, SOD)
			Activation of counter-regulatory mechanism that limits the pro-inflammatory response.	Increased expression of anti-inflammatory cytokines (IL-1RA, IL-10R) and reduced expression of pro-inflammatory genes (TNFSFR).
			Repression of immune cells and host defence, including antigen presentation by phagocytes.	Reduced expression of genes associated with T cells, cytotoxic lymphocytes and natural killer cells (T cell receptor, CD86, IL-2 receptor, TNFRSF7, CD160, cathepsin, CCR7, CXCR3, CD80). Reduced expression in MHC class II genes.
**Calvano **[10]	Early Sepsis (0 to 24 hrs)	↓ more than 1,857 (>50%)^¶^	Activation of host response to infection.	Increased expression of genes associated with pro-inflammatory cytokines (TNF, IL-1, IL-1A, IL-1B, IL-8, CXCL1, CXCL10).
				Increased expression of genes associated with superoxide-producing activities and cell-cell signalling.
			Activation of counter-regulatory mechanism that limits the pro-inflammatory response.	Increased expression of genes that limit the inflammatory response (SOSC3, IL1-RAP, IL1-R2, IL10 and TNFRSF1A).
			Repression of immune cells and host defence, including antigen presentation by phagocytes.	Reduced expression of genes associated with immune response in lymphocytes (TNFRSF7, CD86, CD28, IL-7R, lL-2RB).Reduced expression in MHC class II genes.
**Prabhakar **[9]	Early Sepsis (0 to 24 hrs)	↑31 genes and ↓23 genes	Activation of host response to infection.	Increased expression of pro-inflammatory genes (IL-1B, TRAIL) and S100 calgranulins. Increased expression of genes associated arachidonate metabolites (ALOX5, SOD).
			Activation of counter-regulatory mechanism that limits the pro-inflammatory response.	Increased expression of genes associated with cytokine suppression (SOCS1, SOCS3).
			Reduced antigen presentation by phagocytes.	Reduced expression in MHC class II genes.
**Prucha **[14]	Late-sepsis (1 to 5 days)	↑19 genes and ↓31 genes	Diminished pro-inflammatory response.	Increase expression of pro-inflammatory genes (IL-18, S100A8, S100A12), but reduced expression in others (TNF, IL8RA, CASP5, IL-6ST).
			Enhanced anti-inflammatory response.	Increased expression of anti-inflammatory genes (TGFβ1).
			Reduced lymphocyte function and antigen presentation by phagocytes.	Reduced expression of genes associated with lymphocyte function (IL-16, CD69, CD8, CD36, CX3CR1). Reduced expression in MHC class II genes.
**Tang-1 **[15,16]	Late-sepsis (1 to 5 days)	↑35 genes and ↓15 genes	Diminished pro-inflammatory response.	Reduced expression of pro-inflammatory genes (TNF, IL8RA, CASP5)
			Reduced immune cell function.	Reduced expression of genes that modulate immune cell activation (IL-16, CD69, CD8, CD36).
**Tang-2 **[18]	Late-sepsis (1 to 5 days)	↑105 genes and ↓33 genes	Diminished pro-inflammatory response.	Reduced expression of pro-inflammatory genes (TNFSF8), S100 calgranulins S100A8) and IL-4 pathway.
			Increased anti-inflammatory response.	Increased expression of anti-inflammatory genes (IL-10RB, TGFβ1).
			Reduced antigen presentation by phagocytes.	Reduced expression in MHC class II genes.
**Wong **[23-26]	Late-sepsis (1 to 5 days)	↑862 gene and ↓1,283 genes (Day 1)	Activation of both pro-inflammatory and anti-inflammatory response.	Increased expression of both pro-inflammatory (IL-1 and IL-6) and anti-inflammatory (IL-10, TGFβ1) genes. Increased expression of genes associated with receptor signalling and granulocyte colony stimulating factor.
		↑1,072 gene and ↓1,432 genes (Day 3)	Repression of immune cells and host defence, including antigen presentation by phagocytes.	Reduced expression of genes associated with antigen presentation, immune cell activation, IL-8 and IL-4 pathways.
				Reduced expression in MHC class II genes.
**Cobb **[20,21]	Late sepsis (1 to 5 days)	1,837 genes	Unclear as only a small subset of genes are available for analysis.	Increased expression of pro-inflammatory genes (IL-1beta, NAIP, CEACAM8, and the alpha-defensins).
**Payen **[19]	Recovery (>5 days)	↑1 gene and ↓3 genes (survivors).	Ongoing immuno-suppression throughout the 28-day study period.	In survivors, there was a progressive reduction in the expression of genes associated with S100 calgranulins (S100A8 and S100A12) and T cell activation (IL-3RA).
		↑29 gene and ↓7 genes (non-survivors).	Greater extent of immuno-suppression in non-survivors.	In non-survivors, there was an even greater reduction in the expression of genes associated with immune cell activation (CXCL14, CD180, CD244, CCR6 and CD84). In the same patients, there was also an increase expression of apoptosis genes (PPARG, DAP3 and HBXIP) and anti-inflammatory genes (PAFAH1B1 and IL-4R).
			Survival is accompanied with recovery of some immune functions.	Recovery of MHC class II gene (CD74) in survivors occurs on day 28.
**Pachot **[22]	Recovery (>5 days)	↑18 genes (survivors) and ↑10 genes (non-survivors)	Survival in sepsis is associated with restoration of immune function.	In survivors, there was an increased expression of genes in modulating T cell activation and receptor signalling (ILRB2, CXC31, TRDD3, TIAM1, FYN).

↑ denotes increased gene-expression compared to controls; ↓ denotes reduced gene-expression compared to controls. ^¶^Exact number not given by the author.

We next identified, in each cohort, genes that are well known in the sepsis literature (for example, tumour related factor (TNF), interleukin (IL)-1, IL-8, IL-10 and TGF-beta). In particular, we were interested to see whether there was any systematic difference in their expression patterns between cohorts (for example, early sepsis vs. late sepsis). We restricted our analysis to cohorts of comparable microarray platforms (for example, Affymetrix) and target tissues (for example, whole blood). In this analysis, we found no consistent pattern of gene expression in any of the well-established markers of inflammation (pro-inflammatory or anti-inflammatory). Further analyses by stratifying cohorts based on patient groups (paediatric vs. adults) or presentation (pneumonia or non-specified sepsis) yielded similarly negative findings.

### Experimental sepsis

A major limitation of the above studies is that the findings could be confounded by the variable time from onset of sepsis (since the precise time of infection is often unknown). We, therefore, performed a separate analysis on studies that used an *in vivo *endotoxin challenge model. In these studies, endotoxin was injected into healthy volunteers and blood sampling was performed at regular intervals (up to 24 hours). Consequently, the exact time of onset of infection is known and the effect of timing on gene-expression changes can be clearly defined. We found three endotoxin challenge studies in our data set [8-10]. All three studies used similar experimental protocols. The analysis showed that endotoxin challenge elicited an activation of pathogen recognition and signal transduction pathways, similar to findings in other non-endotoxemia studies. However, the findings on the inflammatory markers were again conflicting. In one study, a predominantly anti-inflammatory profile was observed [8]. In the other two studies, a mixed profile (anti-inflammatory and pro-inflammatory) was observed [9,10]. Hence, even after allowing for the effect of timing, we still could not find any discernible pattern in inflammation-related genes as described in the classic sepsis model.

## Discussion

Historically, cytokine studies suggested that there was a linear transition from pro-inflammatory cytokines to anti-inflammatory cytokines during the course of sepsis. However, these patterns are infrequently seen in clinical settings. In fact, only a few infections follow the classic two-phase model (for example, meningococcal sepsis or contaminated blood transfusions). Recently, studies have shown that inflammatory cytokines in sepsis follow a variable time course [2,3]. Our systematic review extends this growing body of evidence by adding genome-wide data from a variety of clinical settings. In our review, we found that neither a distinctive pro/anti-inflammatory phase nor a clear transition from a pro-inflammatory to anti-inflammatory phase could be seen during sepsis. We also did not observe any discernible pattern in the behaviour of well-established inflammatory markers (for example, TNF-related genes) across the cohorts. Overall, we did not find strong genomic evidence that supports the classic two phase model of sepsis.

The negative finding of our review on the inflammation-related genes is unexpected, considering that the other two well-studied biological phenomena in sepsis, namely the activation of pathogen recognition (for example, toll-like receptors) and signal transduction pathways, are confirmed in most cohorts. The negative finding on inflammation related genes remained even after the cohorts were stratified by timing, patient groups or clinical settings.

The lack of clinical evidence to support the classical two-phase model has been known to many clinicians. The temporal relationship of an early pro-inflammatory phase followed by an anti-inflammatory phase, as depicted in the classical model, is rarely seen in clinical settings. However, this model remains the reigning paradigm under which many anti-sepsis drugs are being developed. The data outlined above therefore provide molecular evidence to validate the increasing concern among clinicians that the current inflammation-based definition of sepsis is too simplistic to describe a complex syndrome [11-13].

While we did not find evidence to support the inflammation-based model of sepsis, we are not able to rule out the existence of other evidence that may support such a model. This is because of the limitations of our study. For example, our review has excluded other gene-expression studies that did not use microarray platform. As a result, our review is based on data from one particular methodology. Studies using other experimental approaches may repudiate/strengthen our findings. Furthermore, the observed gene-expression changes are restricted to circulating leukocytes. The changes in resident leukocytes in local tissue are likely to be very different from circulating leukocytes. Additional data from resident cells will provide a more complete understanding of the host response to sepsis. Another limitation is that our review does not provide information on changes occurring on a proteomic level, as they are not within the scope of this review. Lastly, most studies did not provide information on the leukocyte differential in the blood sample. The variability in leukocyte differentials could have confounded our findings. Given these several limitations, our findings need to be interpreted with caution. A more thorough evaluation of the sepsis model should involve integrating data from other experimental approaches, including *in vitro *studies, animal models and proteomic data.

Our review also revealed several significant methodological limitations of the current microarray studies in sepsis. First, many of the studies included in our review did not make their raw data publicly available. This makes it difficult for other researchers to verify their findings or to undertake meta-analysis. In addition, each study uses different statistical analysis approaches. In particular, different variance estimation methods were used by studies. However, most studies have adequate sample size; hence the impact of variance estimation on our findings is likely to be minimal. Another notable problem is that authors of each paper present their findings differently, making comparison or generalization of their data difficult. For example, some studies reported only a subset of the discovered genes, while others report functional analyses findings without actually listing the discovered genes. To better utilize the findings derived from gene-expression studies of sepsis, a uniform standard of reporting published microarray findings, such as those required for cancer studies [6], should be considered by all study authors in the future.

## Conclusions

Our systematic review shows that sepsis-related inflammatory changes are highly variable on a transcriptional level. The arbitrary distinction of separating sepsis into pro-inflammatory and anti-inflammatory phases is not supported by gene-expression data.

## Key messages

• Sepsis-related inflammatory changes are highly variable on a transcriptional level.

• These changes are not consistent with the established model of sepsis, where a biphasic pro-inflammatory and anti-inflammatory process is thought to underpin the host response.

## Abbreviations

CREB: cAMP responsive element binding protein; JAK: Janus kinase; MAPKs: mitogen activated protein kinase; NF-ĸB: nuclear factor kappa-B; STAT: transducer and activator of transcription protein; TLR: toll-like receptor.

## Competing interests

The authors declare that they have no competing interests.

## Authors' contributions

BT conceived of the study, collected data, performed analyses and drafted the manuscript. BT, SH and AM interpreted the data. All authors read and approved the final manuscript.
